# Advantages of External Reflection and Transflection over ATR in the Rapid Material Characterization of Negatives and Films via FTIR Spectroscopy

**DOI:** 10.3390/polym14040808

**Published:** 2022-02-19

**Authors:** Diego Estupiñán Méndez, Thorsten Allscher

**Affiliations:** Bayerische Staatsbibliothek, Ludwigstraße 16, 80539 Munich, Germany; diego.mendez@bsb-muenchen.de

**Keywords:** material identification, FTIR spectroscopy, cellulose derivatives, transparent plastics, historical films, non-invasive methods, cultural heritage

## Abstract

The identification of film support material is of utmost importance for evidence-based collection management in cultural heritage institutions, especially the identification of cellulose nitrate for fire safety reasons, as nitrate is highly flammable and deteriorates over time. Cellulose nitrate film was used by photographers and movie filmmakers from its release in the 1880s to the 1950s. Cellulose acetate, being called safety film, gradually began to replace cellulose nitrate, as it is not flammable. Despite its non-flammable properties, cellulose acetate also deteriorates in hazardous ways. Therefore, identification of cellulose nitrate and cellulose acetate in collections is imperative for preservation and risk management to collections and humans. Large photographic collections can easily contain several thousand negatives or more, so a rapid, non-invasive and reliable method is needed. Traditional identification methods, such as destructive chemical tests, are sometimes unreliable, and spectroscopic analyses are normally time-consuming. To overcome these issues, rapid material characterization was performed in transflection mode with a Fourier-transform infrared (FTIR) spectrometer equipped with an external reflectance module and an additional aluminum-foil reflector. With this newly developed method, the support material (cellulose nitrate, cellulose acetate and polyester) of about 99.8% of all films can be determined within two seconds of measuring time, without any further spectral processing. Very distinctive spectral patterns are obtained with this new method, regardless of which side of the film is being analyzed. A simple visual inspection of the raw spectrum is usually sufficient to determine the film support identity. A detailed comparison of the various FTIR techniques shows the advantages of the transflection measurement for the material characterization of film support layers. This newly developed method enables the non-invasive, rapid and unambiguous material identification of even large film collections in a short time.

## 1. Introduction

A detailed analysis, or at least a basic determination, of materials is essential for evidence-based collection management in cultural heritage institutions, concerning informed decisions on specific preservation requirements, conservation treatments or storage/exhibition environments. With the acquisition of the STERN image archive in 2019, the Bavarian State Library has become one of the largest picture archives of a public institution in German-speaking countries, with a total of 17.3 million individual images and negatives [1]. To face this huge amount of photographic material, traditionally used procedures for material identification, such as spot testing or the diphenylamine test [2], reach their limits. Moreover, these tests are neither non-invasive nor contactless, but alter the object at the site being examined. The search for spectroscopic techniques for polymer identification led to advances in the application of near-infrared (NIR) spectroscopy [3], NIR chemical imaging [4,5] and organic elemental analysis [6]. Terahertz time-domain spectroscopy was also proposed as a non-invasive method for identification of semisynthetic polymers, such as cellulose nitrate and other cellulose esters [7]. A more common method to analyze photographic materials is the application of Fourier-transform infrared (FTIR) spectroscopy. Especially in the field of cultural heritage, a new generation of commercial FTIR instruments became well established in the last decade. Identification of photographic material by established attenuated total reflection (ATR) spectroscopy has been extensively published, and various photographic techniques, such as albumen, collodion on paper or silver–gelatin have been examined in detail [8,9,10]. In addition, these instruments do not only provide the ATR mode, but also offer various external reflection (ER) techniques for contactless measurements [11,12,13,14,15].

**Figure 1:**
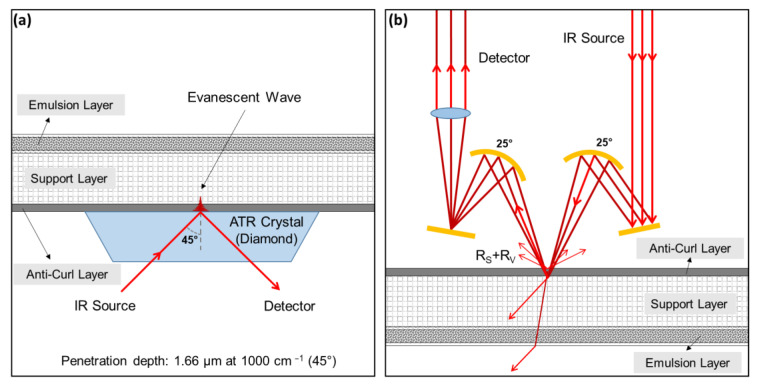
Schematic representation of the both FTIR modes of the Bruker Alpha II: analysis of film material by (**a**) attenuated total reflection (ATR) and (**b**) external reflection (ER).

While these techniques are adequate for the identification of historic polymers in most objects, photographic film represents a significant challenge for infrared spectroscopy due to both sides of the film support commonly being covered with layers of gelatin: silver halide emulsion on the front side and anti-curl layer on the back [16]. The presence of gelatin coatings is likely to interfere with most non-invasive methods, such as FTIR and NIR. The application of ATR spectroscopy only provides information about the material composition of the first approximately 2 µm of an examined surface in contact with the ATR crystal; the deeper layers of a material are hardly reached by the incident IR beam (Figure 1a). To reach these deeper material layers, external reflection is a conceivable and truly non-invasive and contactless alternative measurement (Figure 1b). However, the penetration depth and intensity of reflection of the IR beam depends on various factors, and a part of the IR beam emerges on the transparent back of the sample without contributing to the resulting spectrum [15]. In search of a rapid and definitive technique for the material characterization of negatives and films via FTIR spectroscopy, transflection using an aluminum foil reflector was applied for the first time.

### Principles of Reflection IR Spectroscopy and Instrumentations

With reflection techniques, the intensity of the radiation reflected by the sample surface is reported versus the frequency or wavenumber at the desired angle of detection. As previously described by several authors [15,17], there is an issue with the nomenclature used in reflection spectroscopy. The terms “external reflection” and “diffuse reflection” are used to indicate the different degree of penetration of the IR beam in the medium. For external reflection, the radiation does not penetrate the medium prior to being reflected, while for diffuse reflection, the radiation has penetrated the front surface. However, the term “diffuse” is also used in a strictly geometrical sense to differentiate the IR beams, which are not reflected at a specular angle. In addition, external reflection case by case refers to techniques that collect the radiation reflected from the sample surface, in contrast to total internal reflections, such as ATR, where the electromagnetic radiation undergoes internal reflections in a crystal element. To clarify the use of terms, a short overview of the most relevant reflection principles for the results discussion follows.

In the cultural heritage field, Fabbri et al. [18,19] introduced mid-IR reflectance spectroscopy for the analysis of opaque or partially absorbing objects by the application of mid-IR fiber optics coupled with a bench system and also gave a comprehensive review of the necessary theoretical aspects and definitions for surface reflection (R_S_), volume reflection (R_V_) and total reflectance (R_T_). When opaque or partially absorbing objects are analyzed with FTIR reflectance devices, both R_S_ and R_V_ contribute to the resulting R_T_ spectra. R_S_ and R_V_ depend not only on the optical layout of the measuring system but, primarily, on the analytes’ properties including surface texture, concentration, and IR optical constants, mainly refractive (n) and absorption (k) indexes [20].

According to Fabbri et al. [18,19], R_S_ and R_V_ show an angular distribution which includes specular and diffuse components. R_S_ comprises not only the specular reflection defined as light reflected at the same angle of incidence but can also contain surface reflected light coming from angles different from the incident one. Likewise, R_V_ can emerge from the sample, with an angle of reflection equal to the incident one, as well as coming from different angles, diffusely reflected by the sample. In this latter case, the IR beam penetrating into the sample passes through numerous reflections and refractions events, and, as a consequence, it scatters from numerous points over a wide angle. In summary, the difference of the specular and diffuse components between R_S_ and R_V_ is essentially on the degree of penetration, due to the optical properties of the surface. Rougher and matte textures mostly generate diffuse reflection, while optically flat and shiny surfaces provide a greater amount of specular reflection.

With regard to the FTIR devices, the geometrical layout of the detection system determines the collection of the specular and/or diffuse components, while the extent of both R_S_ and R_V_ depends on the analytes’ properties. In the external reflection configuration, both R_S_ and R_V_ components of the specular and diffuse reflected radiation are detected (see Figure 1b) [11].

The ER spectra differ significantly from those acquired in ATR modes. ER measurements can present large distortions in the spectra with changes in maximum, shape and relative intensity of the IR bands. This is because both R_S_ and R_V_ lead to spectral distortions and the resulting reflection shapes are modeled according to the extent of the R_S_ and R_V_ contributions, which generally co-exist in reflectance IR measurements. The R_S_ gives rise to derivative-like spectral features and/or inverted bands (reststrahlen effect) for absorption bands with absorption k < 1, following the anomalous dispersion of n and for strong oscillators with absorption k >> 1 (strong absorbance coefficient), respectively [17,21,22,23]. Thus, R_S_ is responsible for the main spectral distortions that appear in the reflectance spectra according to Fresnel’s law [15,18].

The R_V_ is basically originated from absorption processes, and it gives similar spectra in terms of shape to those collected in transmission mode. Distortions such as broadening and change in the relative band intensities are usually observed [17]. The penetration depth of the R_V_ diffusely reflected is inversely proportional to both absorption and scattering coefficients [24]. Stronger scattering and absorbing coefficients result in shorter penetration depth, while the penetration becomes almost infinitive for very small k coefficients. For weak absorbers, the IR radiation penetrates deeper, and the repeated refraction and absorbance events enhance the intensity of their IR bands relatively compared to the bands of strong absorbers [15,24].

Other factors can also influence the relative intensities of the bands in diffuse R_V_. At low frequencies, the beam penetrates deeper and, here, lower scattering coefficients occur [25,26]. Therefore, enhancements in the relative intensity for low wavenumber bands relatively to conventional transmission FTIR spectra are usually observed. Overtones (integral multiplies of fundamental absorption frequencies) and combination bands (addition and subtraction of fundamental absorption frequencies) can be found in IR reflectance spectra [27]. Usually overtones and combination bands are weakly intense (small k coefficients), and an increase in their relative intensity can be observed by measuring a longer travel distance in the sample [15].

The spectral features arising from R_S_ and R_V_ can be treated with Kramers–Kronig (KK) operation [28] and the Kubelka–Munk (KM) correction [25]. Both methods can give reliable results when applied to spectra obtained on surfaces where R_S_ and R_V_ predominate. Because of the nature, morphology and heterogeneity of the materials found in cultural heritage objects, R_S_ and R_V_ coexist in the IR reflectance spectrum in unknown variable proportions. This makes the spectra interpretation not straightforward and the use of KK and KM limited [15].

## 2. Materials and Methods

### 2.1. Samples and Chemicals

Nine historical negative films of different support material composition from the photographic collection of the Bavarian State Library were used as exemplary samples.

Polyester vertical A-S Pages in format A4 were purchased from Secol, Ltd., and used as PET reference. Zapon Lacquer, a cellulose nitrate-based varnish diluted in alcohol, a mixture of 1-butanol (15–20%), 2-butoxyethanol (<5%), 2-propanol (<3%) and n-butyl acetate (5–10%), was purchased from Kremer Pigmente (Art. Nr. 79550) and used as cellulose nitrate standard.

### 2.2. FTIR Spectroscopy

ATR spectra were recorded on a Bruker ALPHA II compact FTIR spectrometer that was equipped with a Platinum ATR QuickSnap^TM^ module featuring a monolithic diamond crystal. The penetration depth is 1.66 µm at 1000 cm^−1^, with an incidence angle of 45°. The spectra were obtained in absorbance mode by co-addition of 24 scans with a resolution of 4 cm^−1^, over the range from 4000 to 400 cm^−1^. Before measurements, a background spectrum without sample was collected. Conversion of ATR spectra to absorbance spectra was applied.

For collection of ER spectra, a Reflection QuickSnap^TM^ module was employed, working at an incidence angle of 25°. Background spectra were recorded by using a reference cap with a gold-coated mirror, and sample spectra were recorded by using a sample cap aperture of 3 mm. Both background and sample spectra were obtained in reflectance mode by co-addition of 64 scans with a resolution of 4 cm^−1^, over the range 7500–360 cm^−1^. The raw reflection spectra were processed by using the Kramers–Kronig (KK) transformation tool available in the OPUS software from Bruker Optics (Version 7.5) to obtain pseudo-absorption spectra. Unless otherwise stated, the KK algorithm was applied in the spectral region of 4000–360 cm^−1^.

Transflection spectra were acquired under the same conditions as described for reflection spectra, with the addition of holding a layer of commercially available aluminum foil behind the sample during analysis.

The historical photographic samples were analyzed in ATR, ER and transflection mode on both the back side (image side) and the front side (non-image side) of the film. All FTIR measurements were performed in triplicate (three different areas of the film sample were analyzed, and the resulting spectra were averaged). Unless otherwise stated, the spectra were collected from transparent areas (without image) of the historical films.

For the fast determination of film composition, transflection spectra were visually assessed in the preview mode of the OPUS software prior to scan recording. With this mode, a single scan of the sample can be seen on display before the actual measurement takes place. The display limits in the preview mode were set at 4000–400 cm^−1^ in the x-axis and −0.1–0.5 reflectance units in the y-axis. After acquisition, no further processing was applied to the raw spectra.

### 2.3. SEM Microscopy

SEM micrographs of the film cross-sections were taken without further preparation with scanning desktop electron microscope Phenom ProX (Phenomworld, Eindhoven, Netherlands) G3: Cerium Hexaboride Cathode, SDD-Detector, low vacuum modus.

## 3. Results and Discussion

Nine historical negatives from the collections of the Bavarian State Library (eight films in 35 mm format and one sheet film) were chosen as model samples for their analysis via FTIR spectroscopy (see Table 1, Samples 1–9). The chemical composition of the film support of all nine samples was first determined by ATR, and the results were confirmed via polarization test (positive for polyester, Sample 9) and a minimally invasive version of the burn test [29] (positive for cellulose nitrate, Samples 5–8). Additionally, commercially available polyester sleeves (PET, Sample 10) and Zapon lacquer (cellulose nitrate varnish, Sample 11) were used as reference samples. The main properties of the analyzed samples are summarized in Table 1.

**Figure 2:**
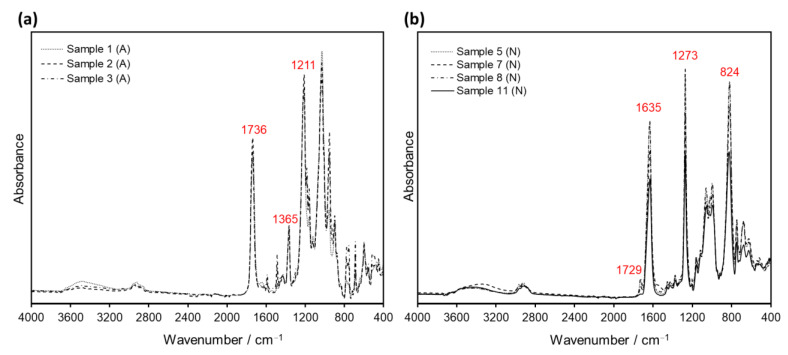
ATR spectra of (**a**) Samples 1–3, identified as cellulose acetate (A); and (**b**) Samples 5, 7, 8 and 11, identified as cellulose nitrate (N). Spectra were collected from the front side (non-image side) of the historical films.

The ATR spectra of cellulose derivatives show characteristic signals from the cellulose backbone, as well as particular bands specific to the acetate and nitrate side groups [30]. The spectra of the cellulose acetate samples analyzed in this study displayed bands at 1736, 1365 and 1211 cm^−1^ corresponding to carbonyl stretching, methyl bending and ester stretching, respectively, as exemplified in Figure 2a for Samples 1–3. On the other hand, the cellulose nitrate samples exhibited characteristic vibration bands at 1635, 1273 and 824 cm^−1^ (for example Samples 5, 7 and 8 in Figure 2b) from the nitrate groups. In both cases, bands corresponding to hydroxyl stretching (3600–3200 cm^−1^), CH and CH_2_ stretching (3000–2900 cm^−1^) and cellulose backbone vibrations (1200–900 cm^−1^) were observed.

Additionally, a reference cellulose nitrate sample was prepared by adding dropwise commercially available Zapon lacquer on a Teflon surface (see Supplementary Figure S1). After complete evaporation of the solvent, Sample 11 was obtained, and its ATR spectrum was compared with the historical Samples 5–8. Except for a small band at 1729 cm^−1^ present in Samples 5–8 and absent in reference Sample 11 (corresponding to camphor, a plasticizer frequently used for cellulose nitrate plastics), the recorded ATR spectra were identical, confirming their classification as cellulose nitrate (see Figure 2b).

The structural information retrieved from ATR spectra allowed a clear distinction between cellulose acetate and cellulose nitrate samples. Nevertheless, the ATR technique presented some drawbacks for its use as an identification tool in large photographic collections of historical value. Frist, to ensure an intimate contact between the ATR crystal and the sample, considerable pressure has to be exerted on the film, and such pressure could leave marks on its surface or even damage very sensitive samples. A second issue arises from the limited penetration depth of the evanescent wave in ATR mode, reaching only a few μm into the sample [9,11,31]. Due to the layered structure of negative films, probing via ATR might not be able to detect the film support in samples with higher optical density.

**Figure 3:**
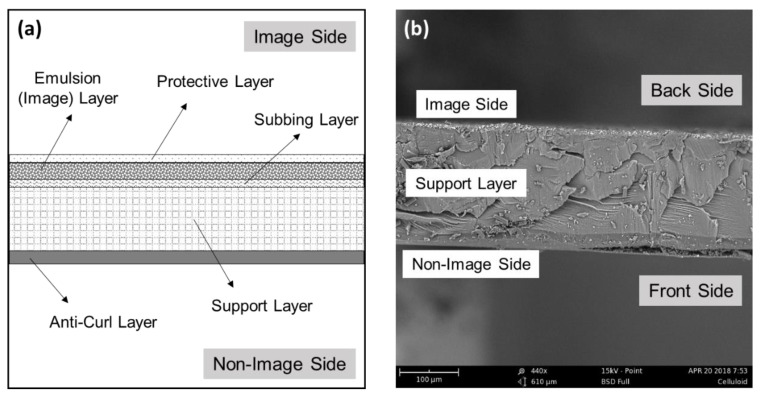
Cross-section of a black-and-white photographic film, showing its different layers, in particular, the gelatin-based protective, emulsion, subbing and anti-curl layers, as well as the polymeric support layer (cellulose nitrate, cellulose acetate or polyester): (**a**) schematic representation and (**b**) SEM micrograph. The film thickness in this example is between approximately 208 and 211 µm, and the thickness of the image layer varies between approximately 6 and 11 µm.

Negative films are structured materials consisting of several layers with different chemical compositions, as depicted in Figure 3. The number and thickness of these layers widely vary depending on the film type and manufacturer. In a simplified way, a black-and-white photographic film comprises an emulsion layer (colloidal silver salts dispersed in gelatin) where the image is formed, a transparent polymeric support (cellulose derivative or polyester) and a subbing layer (a mixture of cellulose derivative and gelatin) to ensure adhesion between emulsion and support. Additionally, antihalation/antistatic layers and protective anti-scratch/anti-curl layers made of clear gelatin are also part of the film structure [16,32] (see Figure 3a). Moreover, color negatives possess at least three different emulsion layers (sensitive to blue, green and red light) and additional filter and spacer layers. This means that the film support will normally be closer to the surface of the non-image side of developed films.

**Figure 4:**
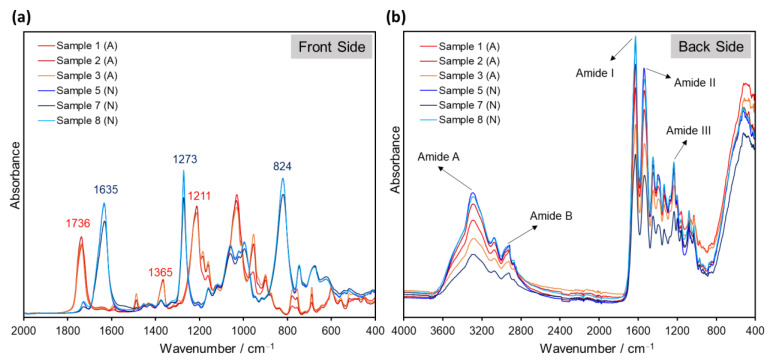
ATR spectra of Samples 1–3 (cellulose acetate, A, red) and Samples 5, 7 and 8 (cellulose nitrate, N, blue) measured (**a**) on the front (non-image) side and (**b**) on the back (image) side of the films.

Accordingly, the spectral features of Samples 1–8 after ATR analysis were found to be strongly dependent on the sample orientation during testing, showing differences for the back (image) side and the front (non-image) side of the films. On the one hand, the front-side spectra displayed the characteristic signals of the support material, permitting its identification (either cellulose acetate or cellulose nitrate; see Figure 2 and Figure 4a). On the other hand, ATR spectra of the same samples taken from the back side of the film exhibited signals typical for gelatin, plausibly from the protective and/or emulsion layers. In fact, the bands Amide A (3292 cm^−1^), Amide B (2922 cm^−1^), Amide I (1626 cm^−1^), Amide II (1537 cm^−1^), Proline/Hydroyxproline (1448 cm^−1^) and Amide III (1236 cm^−1^), characteristic of gelatin [33], were observed in the back side-ATR spectra of all samples regardless of their different film support (see Figure 4b).

**Figure 5:**
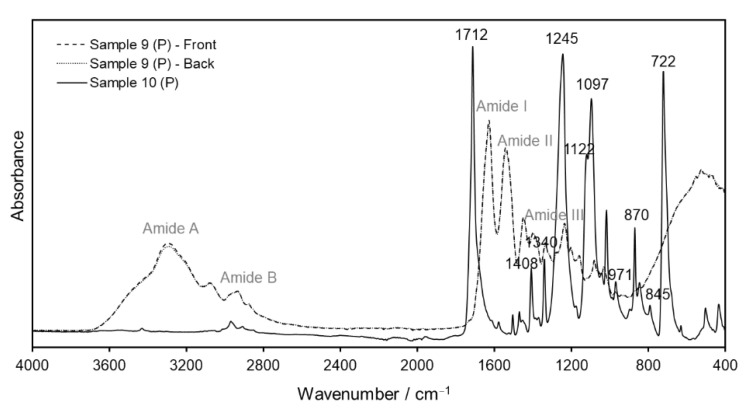
ATR–IR spectra of Sample 10 (PET sleeve, solid line) and Sample 9 (polyester, P) taken on the front side (dashed line) and the back side (dotted line) of the film.

Evidently, the ATR evanescent wave was not able to penetrate into the support layer of the samples for the measurements performed on the back side of the films. In this case, ATR fails to retrieve the structural information required for the identification of the film’s base material. Although, in most cases, this issue can be bypassed by measuring always the front side of the films, this process is time-consuming and, for some samples, it might not even be possible to detect the support material via ATR at all. For example, polyester films normally have protective/anti-scratch layers in both (front and back) sides of the film. Thus, instead of exhibiting the characteristic signals of PET [34], the ATR analysis of Sample 9 provided only bands corresponding to gelatin, irrespective of which film side was measured. Conversely, ATR analysis of the commercial PET sheet (Sample 10) yielded the expected bands for carbonyl stretching at 1712 cm^−1^; C–O stretching at 1408 and 1340 cm^−1^; deformation of the terephthalate residues at 1245 and 1122 cm^−1^; methylene group vibrations at 1097 cm^−1^; and aromatic ring vibrations at 971, 870 and 845 cm^−1^ (Figure 5).

**Figure 6:**
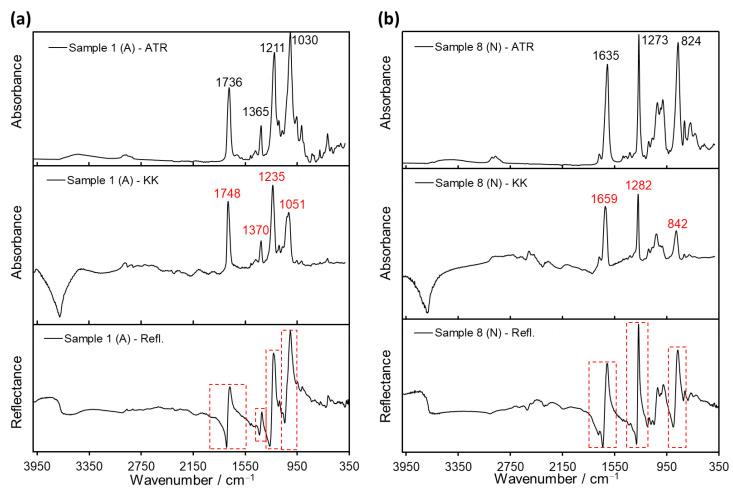
Comparison of raw ER spectra (bottom), their respective pseudo-absorption spectra calculated after KK transformation (middle) and ATR spectra (top) of films: (**a**) Sample 1 (cellulose acetate, A) and (**b**) Sample 8 (cellulose nitrate, N). The derivative-like bands in the reflection spectra (red dashed lines) are transformed into absorption-like bands by the KK algorithm and resemble the absorption bands in the ATR spectra. Both reflection and ATR spectra were recorded from the front side (non-image side) of the historical films.

An alternative non-invasive method to ATR is ER spectroscopy. Samples 1–11 were analyzed with the external reflection technique provided by the Bruker Alpha spectrometer, and the resulting curves were treated with the KK transformation to compare them with the results obtained from ATR. As exemplified in Figure 6 for Samples 1 and 8, the raw reflection spectra exhibit derivative-like bands, expected for polymer samples. After applying the KK algorithm to the reflection data, absorbance-like bands were obtained at 1748, 1370, 1235 and 1051 cm^−1^ for Sample 1 (cellulose acetate), and at 1659, 1282 and 842 cm^−1^ for Sample 8 (cellulose nitrate). The principal peaks found after KK transformation roughly correspond to the signals of acetate (1736, 1365 and 1211 cm^−1^) and nitrate (1635, 1273 and 824 cm^−1^) side groups determined from ATR (refer also to Figure 2); however, a shift of 10–20 cm^−1^ in the peak position was observed in the KK curves (Figure 6).

Even though the absolute values from ER spectroscopy do not perfectly match the ATR data, these results confirm that KK spectra represent a reliable alternative to ATR for the non-invasive identification of support materials in historical films. Nevertheless, some spectral features obtained via ATR, e.g., residual hydroxyl groups (3600–3200 cm^−1^), CH and CH_2_ stretching bands (3000–2900 cm^−1^) or cellulose backbone vibrations (1200–900 cm^−1^), are not available from KK-operated ER spectra (see Figure 6).

**Figure 7:**
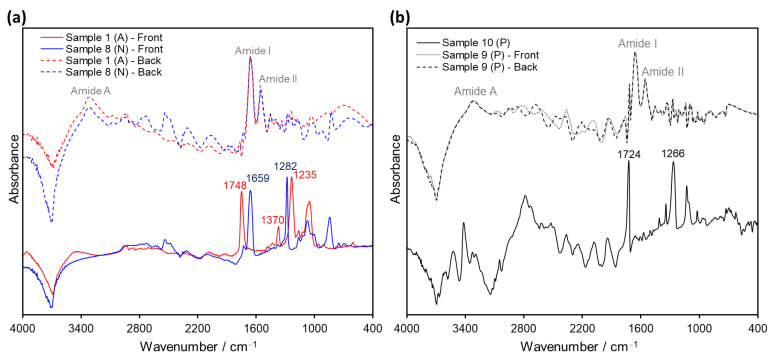
Comparison of KK-operated ER spectra of (**a**) Samples 1 (cellulose acetate, A, red) and 8 (cellulose nitrate, N, blue) determined on the front (solid line) and the back (dashed line) side of the films; and (**b**) Samples 10 (PET sleeve, solid line) and 9 (polyester, P) recorded on the front (dotted line) and back (dashed line) side of the film.

On the other hand, it has been suggested that reflection measurements afford slightly deeper penetration into the sample than the ATR method [31], even though both of them are surface-analysis techniques. Therefore, reflection analyses were carried out on both sides of the sample films in order to investigate the detection capacity for the inner support layer. However, the evaluation of the reflection spectra of Samples 1–8 after KK revealed that only the measurements on the front side permitted the discrimination between cellulose acetate and cellulose nitrate, as exemplified in Figure 7a for Samples 1 and 8, respectively. Conversely, the analysis of the back side of the films yielded only signals corresponding to gelatin (e.g., Amide A at 3315 cm^−1^, Amide I at 1659 cm^−1^ and Amide II at 1555 cm^−1^), as previously observed for ATR. The same issue was encountered for Sample 9 (see Figure 7b), for which neither front nor back side measurements retrieved the expected signals for PET (especially the strong carbonyl and terephthalate signals, which were observed in Sample 10 at 1724 and 1266 cm^−1^, respectively).

**Figure 8:**
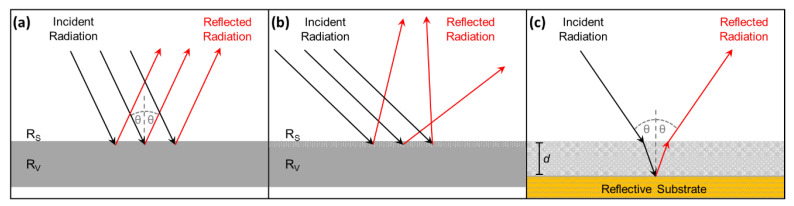
Schematic representation of reflection phenomena: (**a**) specular reflection (only R_S_ component shown), (**b**) diffuse reflection (only R_S_ component shown) and (**c**) transflection.

As mentioned above, both the R_S_ and R_V_ contribute to the resulting ER spectrum depending on the various sample properties. Furthermore, R_S_ and R_V_ exhibit an angular distribution of reflected radiation; that is, light can be reflected either at the same angle of incidence (see Figure 8a) or at different angles (see Figure 8b). The former case is termed “specular reflection” and the latter “diffuse reflection”, both of which can take place at the surface (R_S_), as well as at the volume (R_V_), level of the sample [15,18,21]. Specular reflection is typical for smooth and shiny samples, while diffuse reflection is more relevant for rough samples with good light-scattering properties [25]. Another phenomenon related to R_V_ is the so-called transflection, which occurs when an at least partially transparent sample is placed in front of a smooth reflective substrate or mirror (e.g., a metal surface) [15,21,25]. For transflection, the incident radiation first passes through the volume of the sample until it reaches the metal substrate, where it is specularly reflected and passes a second time through the sample (see Figure 8c). Therefore, the transflection phenomenon usually generates reflection spectra that resemble transmission bands of the analyzed material [21].

**Figure 9:**
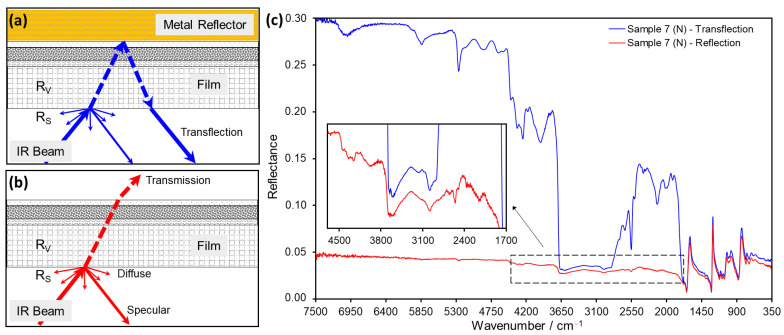
Schematic representation of reflection phenomena in transparent films: (**a**) transflection (R_V_ favored) and (**b**) external reflection (R_S_ favored). Specular and diffuse reflection components are detected in both cases. (**c**) Comparison of transflection (blue) and ER spectra (red) of Sample 7 (cellulose nitrate, N). The spectral bands associated with R_V_ are much more intense in the transflection spectrum than in the ER spectrum (see inset).

The double transmission of incident light through the sample that takes place during transflection increases the intensity of the reflection signals and enhances their R_V_ component [15] (Figure 9). Hence, a typical transflection spectrum consists of derivative-like bands at lower wavenumbers corresponding to R_S_, along with very intense bands associated with R_V_ at higher wavenumbers, as exemplified in Figure 9c for Sample 7, in the spectral regions 1700–600 cm^−1^ (R_S_) and 4500–1700 cm^−1^ (R_V_).

**Figure 10:**
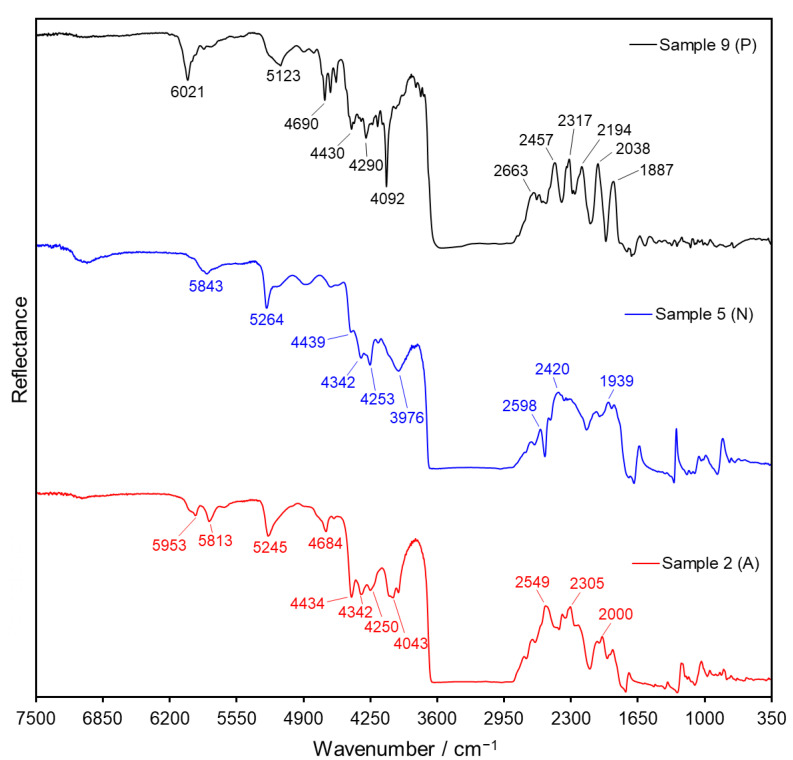
Transflection spectra of Sample 2 (cellulose acetate, A, red), Sample 5 (cellulose nitrate, N, blue) and Sample 9 (polyester, P, black). Spectra were collected from the front side (non-image side) of the films.

The transflection spectra of cellulose acetate, cellulose nitrate and polyester are markedly different (as exemplified in Figure 10 for Samples 2, 5 and 9, respectively), displaying particular signals that permit their identification. The first region of interest extends through the short-wavelength infrared from 6150 to 3600 cm^−1^, in which transmission-like bands can be observed. In this region, the cellulose derivatives display a similar spectral pattern that is distinct from the signals observed in the polyester sample. Cellulose nitrate exhibit broad bands at 3976, 5264 and 5843 cm^−1^, which are also found in the spectrum of cellulose acetate at slightly different positions, namely 4043, 5245 and 5813/5953 cm^−1^ (here two peaks instead of one). The signals at 4250, 4342 and 4435 cm^−1^ are common to both spectra; however, for cellulose acetate, they have the same height, and for cellulose nitrate, they decrease in reflectance intensity from left to right in the spectrum. In addition, cellulose acetate shows a band at 4684 cm^−1^, which is absent in the spectrum of cellulose nitrate. Contrarily, the spectrum of polyester exhibit sharper signals, with the most important of them located at 4092, 4290, 4430, 4690, 5123 and 6021 cm^−1^ (see Figure 10).

However, the most evident differences in the spectra of the film samples arise in the region 2850–1770 cm^−1^. Here, the cellulose derivatives exhibit three broad peaks, located at 2549, 2305 and 2000 cm^−1^ for cellulose acetate and at 2598, 2420 and 1939 cm^−1^ for cellulose nitrate. Moreover, in the spectrum of cellulose acetate, the first two peaks (2549 and 2305 cm^−1^) have similar intensity, and the third one (2000 cm^−1^) is less intense, while for cellulose nitrate, the opposite was observed (the peak at 2598 cm^−1^ has lower intensity than the other two). On the other hand, the spectrum of polyester shows a series of sharp peaks, one of lower intensity at 2663 cm^−1^ and another five more intense ones at 2457, 2317, 2194, 2038 and 1887 cm^−1^ (see Figure 10). Lastly, in the region 1700–500 cm^−1^, the typical R_S_ derivative-like reflection bands of cellulose acetate and cellulose nitrate can be observed (compare to reflection spectra in Figure 6).

The strong signals associated with R_V_ in the transflection spectra of the historical films provide a reliable marker for fast material identification. The determination of film composition can be rapidly achieved by a simple visual inspection of the spectral pattern around 2550 cm^−1^, since cellulose acetate displays a peak, cellulose nitrate a trough and polyester several peaks in this region. Moreover, to assess the difference between these support materials usually one single scan is sufficient. The preview mode of the OPUS software lends itself very well to this purpose. With this mode, a single scan of the sample is displayed and identification of the support material can be performed in less than 2 s, before the actual recording of the spectra takes place. Moreover, neither spectral processing nor mathematical operations are required in transflection mode for the identification of film material. Screenshots of the preview mode visualization of the spectra for Samples 3 (cellulose acetate), 8 (cellulose nitrate) and 9 (polyester) are shown in Supplementary Figure S2, exemplifying the potential of this technique for fast material determination of historical films.

**Figure 11:**
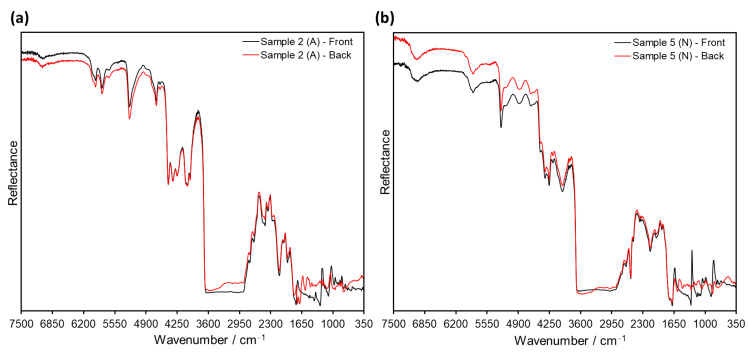
Comparison of transflection spectra of (**a**) Sample 2 (cellulose acetate, A) and (**b**) Sample 5 (cellulose nitrate, N). Measurements were performed on the front (black) and the back (red) side of the films.

Apart from the distinctive spectral patterns achieved via transflection, another advantage of this technique in comparison to conventional ATR and ER measurements is its increased penetration depth. Since in transflection the IR beam passes through the sample twice (see Figure 9a), the inner support layer in the film material can always be analyzed, regardless of which side of the film is being measured. As depicted in Figure 11 for cellulose Samples 2 and 5 (acetate and nitrate, respectively), the spectral features associated with R_V_ (ca. 6500–1700 cm^−1^) are identical for measurements performed on the front (non-image) side and the back (image) side of the films. On the contrary, the bands associated with R_S_ (ca. 1700–700 cm^−1^) have a strong dependence on the side chosen for measurement (gelatin signals for back measurements; cellulose derivative support for front measurements), as observed before in reflection mode (refer also to Figure 7a). The fact that film samples can be analyzed from both sides via transflection not only speeds up the identification process in large photographic collections, but also grants access to structural information of double-coated films. For instance, Sample 9 could not be spectroscopically identified as PET via ATR or ER measurements. These techniques granted access only to the outermost gelatin layers, whose characteristic bands were observed on both sides of the film (see Figure 5 and Figure 7b), contrarily to the distinct polyester signals obtained via transflection for this sample (refer to Figure 10 and Supplementary Figure S3).

**Figure 12:**
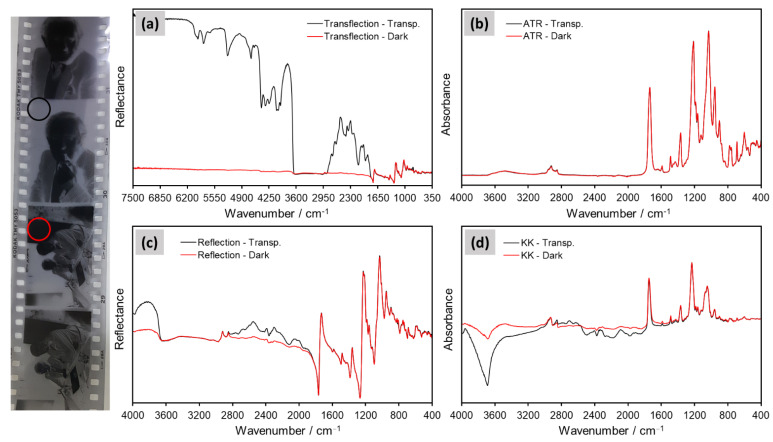
Spectra of Sample 4 (cellulose acetate) recorded from transparent (black curves) and dark (red curves) areas in the film: (**a**) transflection, (**b**) ATR, (**c**) ER and (**d**) KK transformation of the ER data. Measurements were performed on the front (non-image) side of the film, at the spots indicated in the black and red circles.

Nevertheless, a disadvantage of the transflection method in comparison to ER and ATR measurements is that the analyses have to be carried out in transparent areas of the film. As depicted in Figure 9a, during transflection, the incident light must pass through the film to be reflected by the metal surface. If the optical density of the sample is too high, the radiation will be reflected at the surface and will not reach the metal reflector [8]. Film negatives usually have dark optically denser areas where the image is present, in which material identification via transflection is not possible. As shown in Figure 12 for Sample 4 (cellulose acetate), the R_V_ signal enhancement accomplished by transflection was only observed when transparent areas of the film were analyzed. Contrarily, the main signals in the ATR and ER (KK) spectra did not change significantly when dark areas in the film were sampled (Figure 12). In fact, transflection spectra collected from dark areas of the film correspond well to ER spectra recorded from transparent areas of the film. Plausibly, in both cases, the incident light is reflected at the surface of the film (no back reflection) and the R_S_ component dominates the spectra (see Supplementary Figure S4). This issue can be also encountered in degrading films, which exhibit dark staining of the film base, appearance of oxidation marks, decomposition of silver complexes and image discoloration. Such signs of degradation that are present in many film collections increase the optical density of the once-transparent areas of the film and hinder the analysis via transflection spectroscopy.

Notwithstanding, the transflection method is ideally suited to identify support materials in large photographic collections. Compared to ATR, transflection measurements are faster, require less sample handling and avoid applying pressure on the film for its analysis. Compared to external reflection, analyses via transflection can be performed in shorter times (usually one scan is needed; see Supplementary Figure S2), and no further treatment of the spectra (such as KK transformations) is necessary. Moreover, in contrast to ATR and external reflection, transflection spectra can be recorded on either side of the film to analyze the inner support material. In this way, numerous film samples can be analyzed in a fast and reliable fashion via transflection, provided that the films present transparent optically rarer areas. By employing this method, any transparent negative film material can be entirely analyzed. Up to now, almost 480,000 films could be examined by using this method. About 1.8% of the films were identified as cellulose nitrate and 96% as cellulose acetate. For less than 0.2% of the films, the transflection method could not be successfully applied, due to the lack of transparent areas in the sample. In such cases, complementary identification methods (ATR, polarization test, diphenylamine test and burn test) were used to determine the chemical composition of the film support.

## 4. Conclusions

For evidence-based collection management in cultural heritage institutions, the determination of the support material of historical films is essential, especially the identification of cellulose nitrate for fire safety reasons. Traditionally, the identification of film support materials has been carried out by gathering contextual information, such as dates of release of the films, their trade names and physical appearance, or by performing destructive chemical tests. In the last years, these destructive methods for the analysis of films have been gradually replaced by non-invasive spectroscopic techniques. Among them, ATR and external reflection spectroscopy have become the methods of choice for many cultural heritage institutions worldwide. However, these techniques present some drawbacks for the analysis of large collections, such as extensive sample handling, relatively long measuring times, low penetration depth or the post-treatment of the recorded spectra. Such issues limit the wide application of ATR and external reflection measurements, especially for unexperienced users.

In the present study, for the first time, transflection was investigated for 11 model samples as an alternative method for the determination of film support composition. With this new technique, structural information from negative films can be attained in a fast and reliable way from the raw spectrum without further spectral processing. Since radiation is transmitted through the sample before being reflected by a metal surface, transflection spectra are more intense and comprise a higher R_V_ component than conventional reflection spectra, which is advantageous for the analysis of the inner support layer of the films. Hence, very distinctive spectral patterns can be obtained via transflection for cellulose acetate, cellulose nitrate and polyester supports (especially in the range 2850–1770 cm^−1^), regardless of which side of the film is being analyzed. Moreover, a simple visual inspection of the raw spectra in preview mode (one scan) is usually sufficient to determine the film support identity in less than two seconds. Provided that transparent areas are present in the film sample, transflection represent a straightforward methodology for data recording and interpretation. In fact, more than 480,000 negatives from the collections of the Bavarian State Library were analyzed by using the transflection technique, and their identity could be successfully determined. Nevertheless, the transflection phenomenon is hindered in optically denser areas in the sample, such as the silver image in good conserved films, as well as dark stains caused during film degradation. Less than 0.2% historical negatives from the collections were too dark to be analyzed via transflection. In such cases, the analyses were complemented with the traditional methods in order to determine the film composition.

The performed transflection analyses were focused solely on rapid material identification; however, the potential of this technique to retrieve more detailed structural information (e.g., extent of degradation, presence of plasticizers, etc.) remains to be investigated. In addition, a systematic study of the influence of sample thickness on the transflection signals is currently ongoing. Further work is currently in progress to assess the applicability of the transflection method for the analysis of motion picture films, X-Ray films, microfilms and other transparent plastics relevant for conservation in cultural heritage institutions. Finally, transflection mode is applicable to other transparent materials in combination with aluminum foil or any other reflecting material. This contactless and non-invasive method provides spectral information from deeper layers of a material than ATR or external reflection without a reflector.

## Figures and Tables

**Table 1:** Historical and commercial samples selected for FTIR spectroscopic analysis.

Sample	Film Support	Format	Trade Name/Manufacturer
1	Cellulose Acetate	35 mm	Eastman L Panchromatic
2	Cellulose Acetate	35 mm	Agfa L Isopan F
3	Cellulose Acetate	35 mm	Ilford Panchromatic Hypersensitive
4	Cellulose Acetate	35 mm	Kodak TMY 5053
5	Cellulose Nitrate	35 mm	Agfa 409
6	Cellulose Nitrate	35 mm	Agfa Isopan F
7	Cellulose Nitrate	35 mm	Kodak KB17
8	Cellulose Nitrate	35 mm	Zeiss JKON
9	Polyester	Sheet Film	n.a.
10	Polyester ^1^	n.a.	Polyester Pages/Secol Ltd. (Thetford, UK)
11	Cellulose Nitrate ^2^	n.a.	Zapon Lacquer (Springfield, NJ, USA)/Kremer Pigmente

^1^ Commercial PET sleeve. ^2^ Commercial cellulose nitrate varnish.

## Data Availability

The data presented in this study are available upon request from the corresponding author.

## Supplementary Materials

The following supporting information can be downloaded at: https://www.mdpi.com/article/10.3390/polym14040808/s1, photographs of the Zapon lacquer film Sample 11 (Figure S1); screenshots of the preview mode spectra of Samples 3, 8 and 9 (Figure S2); transflection spectra of Samples 9 and 10 (Figure S3); transflection and reflection spectra of Sample 4 (Figure S4); and FTIR spectra of all samples (Figures S5–S15).
