# Supplementary material for: Advantages of External Reflection and Transflection over ATR in the Rapid Material Characterization of Negatives and Films via FTIR Spectroscopy
Source: Polymers (Basel). 2022 Feb 19;14(4):808. doi: 10.3390/polym14040808 (PMC8878977; doi:10.3390/polym14040808)
Supplement: Supplementary file 1 [file polymers-14-00808-s001.zip › polymers-1600710-supplementary.pdf]

Supplementary Data

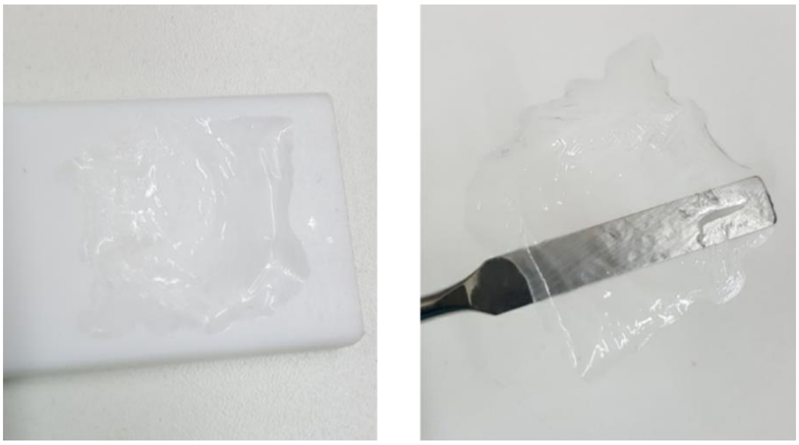

Figure S1. Thin film of cellulose nitrate prepared by successive addition of Zapon lacquer over a Teflon surface. The solvent mixture of the Zapon varnish (1-butanol, 2-butoxyethanol, 2-propanol and n-butyl acetate) was left to evaporate under the fume hood overnight and the resulting film was removed from the Teflon with a spatula to obtain a transparent cellulose nitrate layer (Sample 11).

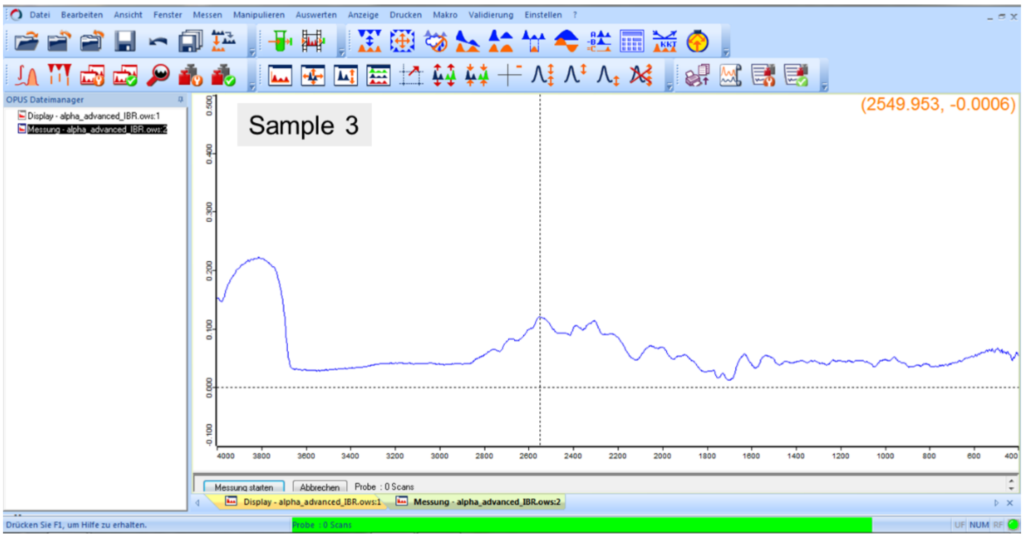

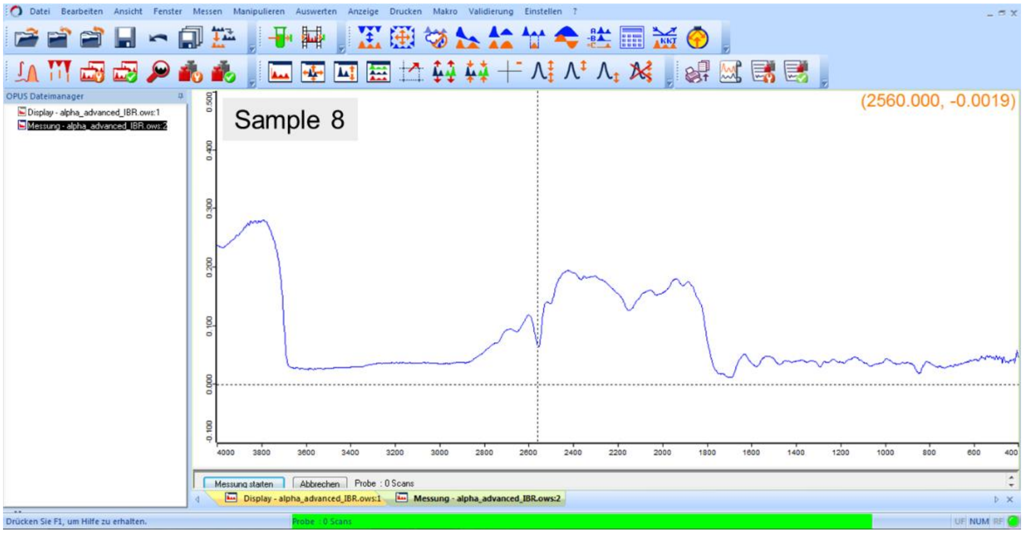

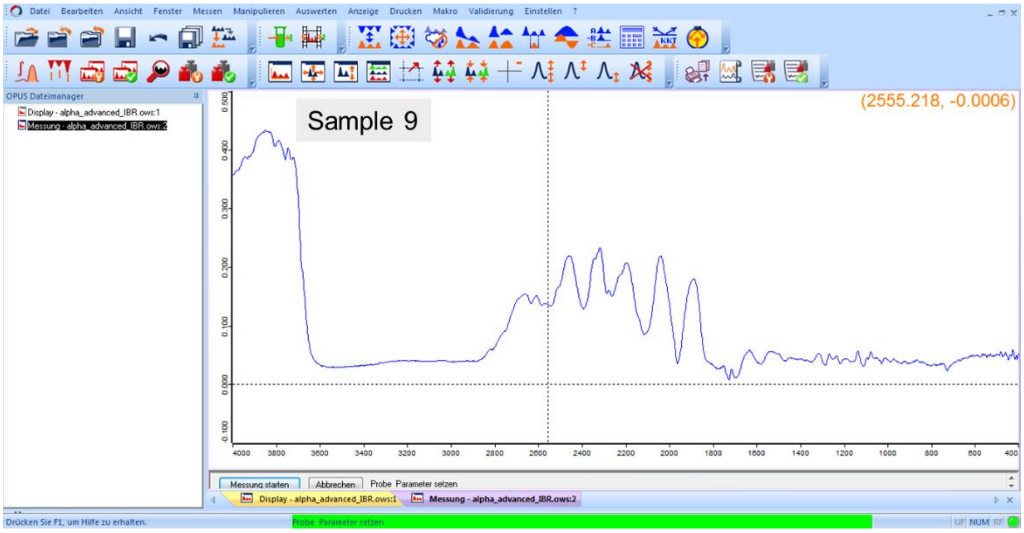

Figure S2. Screenshots of the transflection spectra of historical films Sample 3 (cellulose acetate), Sample 8 (cellulose nitrate) and Sample 9 (polyester), visualized in the preview mode of the OPUS software (one scan). The distinctive spectral features at around 2550 cm^{-1} (one peak for cellulose acetate, one trough for cellulose nitrate several peaks for polyester) can be used as identification criteria for the support material of the films.

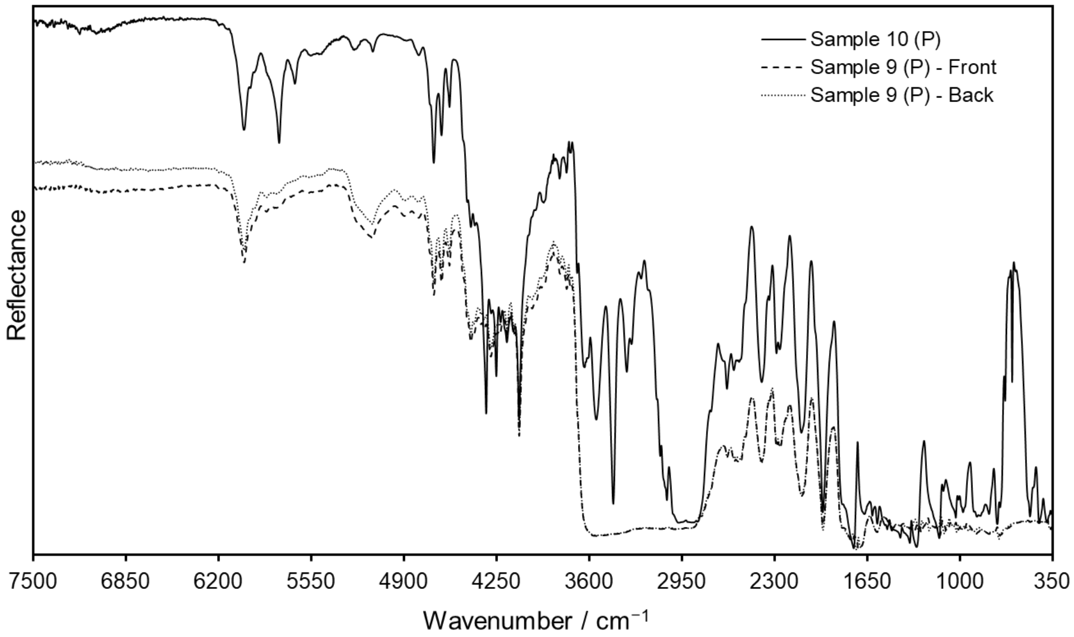

Figure S3. IR-Transflection spectra of Sample 10 (PET sleeve, solid line) and Sample 9 (polyester historical film) taken on the front side (dashed line) and the back side (dotted line) of the film. The R_V signals at 5776, 4324, 4251 and 3647–2951 cm^{-1} are present in the PET sheet but absent in the historical film, probably due to differences in the thickness and internal structure of the samples. The R_S signals at 1750–360 cm^{-1} can only be observed in the PET sheet (Sample 10), since the double gelatin coating of the polyester film (Sample 9) prevents its analysis

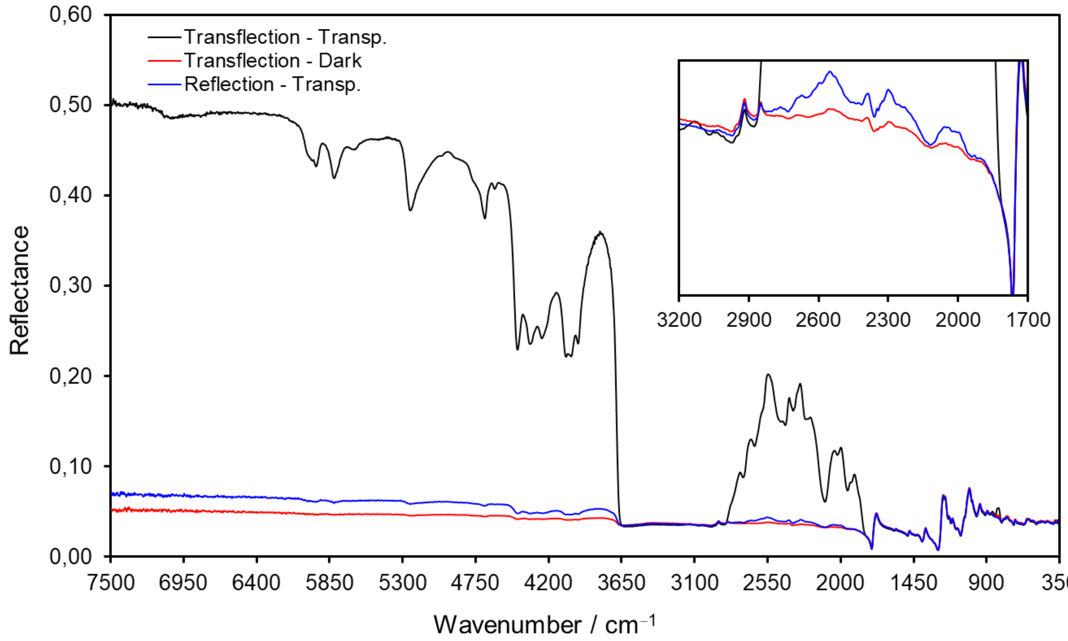

Figure S4. Transflection spectra of Sample 4 (cellulose acetate) recorded from a transparent area (black curve) and a dark area (red curve) in the film. Since incident radiation cannot reach the metal surface at the optically denser dark areas, it is reflected at the film surface and the resulting transflection spectrum resembles the reflection spectrum recorded from a transparent area in the film (blue curve).

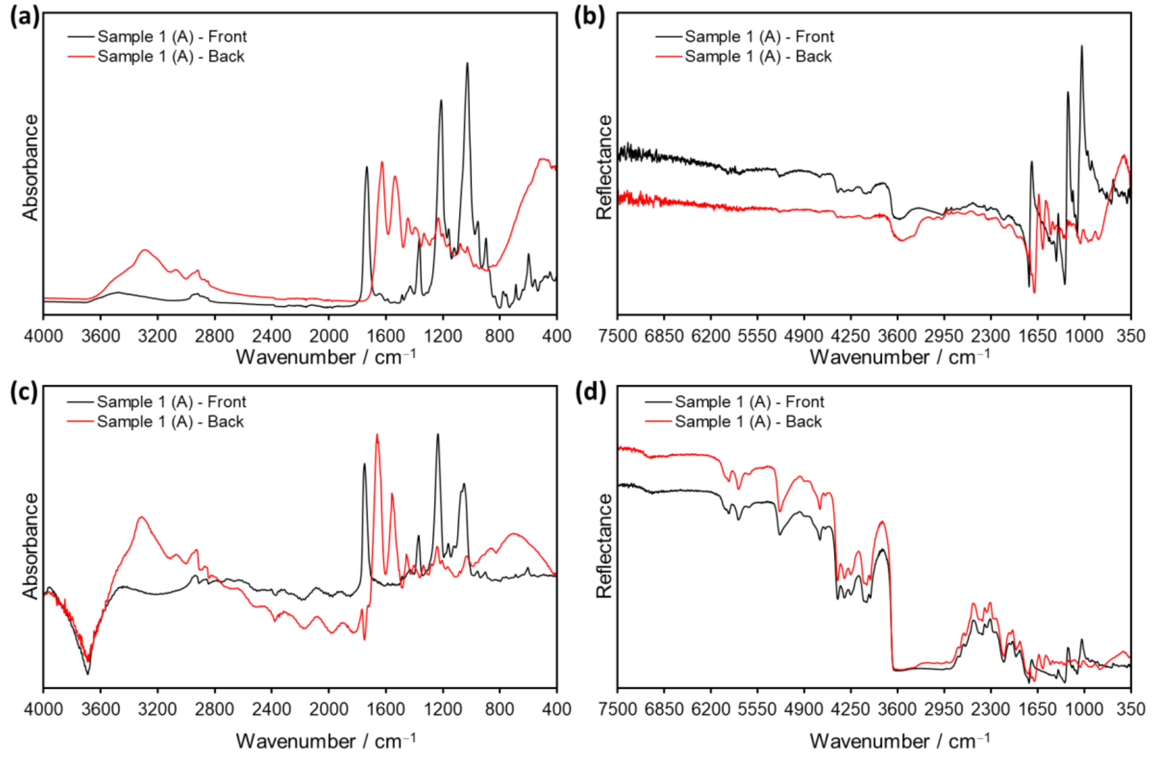

Figure S5. FTIR spectra of Sample 1 (cellulose acetate) recorded on the front side (black) and the back side (red) of the film: (**a**) ATR; (**b**) reflection; (**c**) KK transformation of reflection curves; (**d**) transflection.

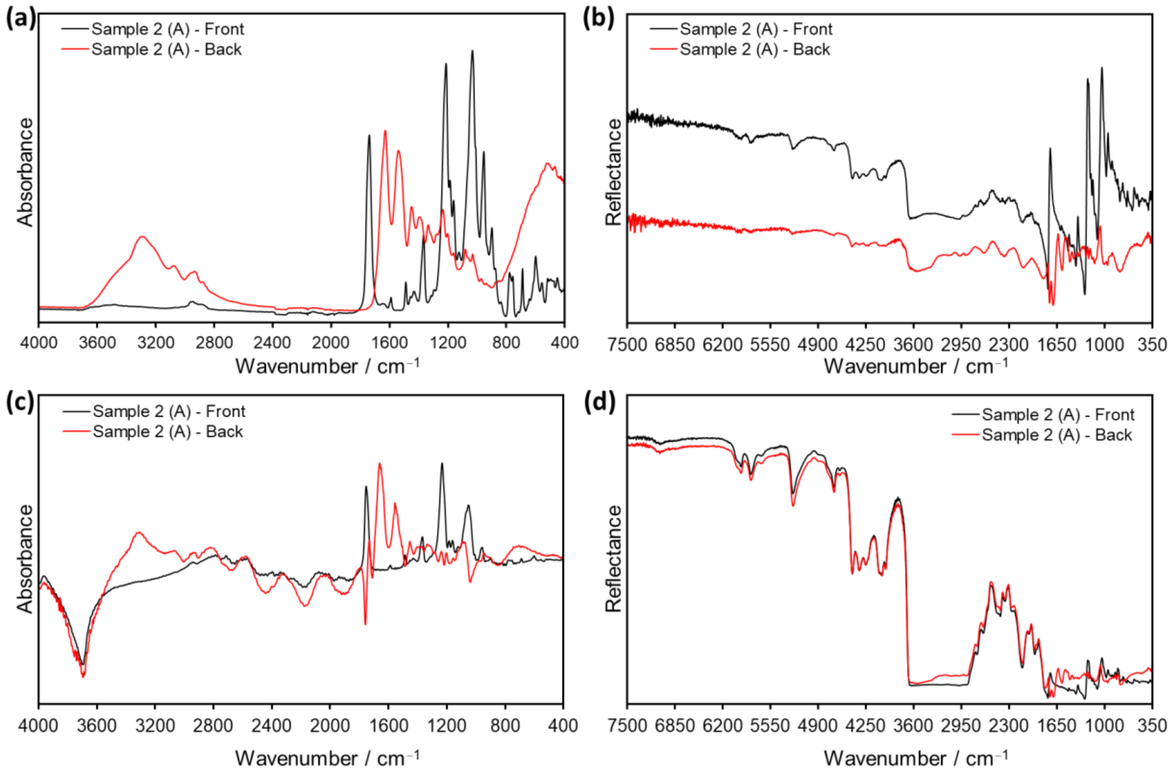

Figure S6. FTIR spectra of Sample 2 (cellulose acetate) recorded on the front side (black) and the back side (red) of the film: (**a**) ATR; (**b**) reflection; (**c**) KK transformation of reflection curves; (**d**) transflection.

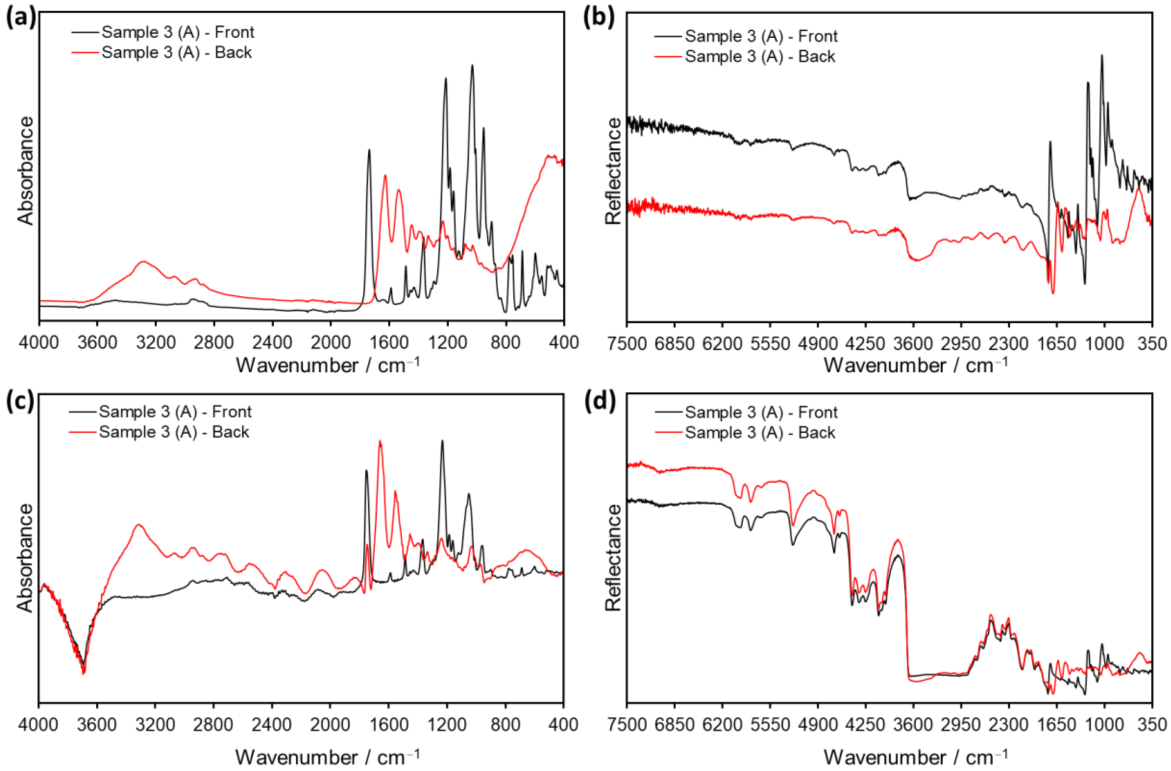

Figure S7. FTIR spectra of Sample 3 (cellulose acetate) recorded on the front side (black) and the back side (red) of the film: (**a**) ATR; (**b**) reflection; (**c**) KK transformation of reflection curves; (**d**) transflection.

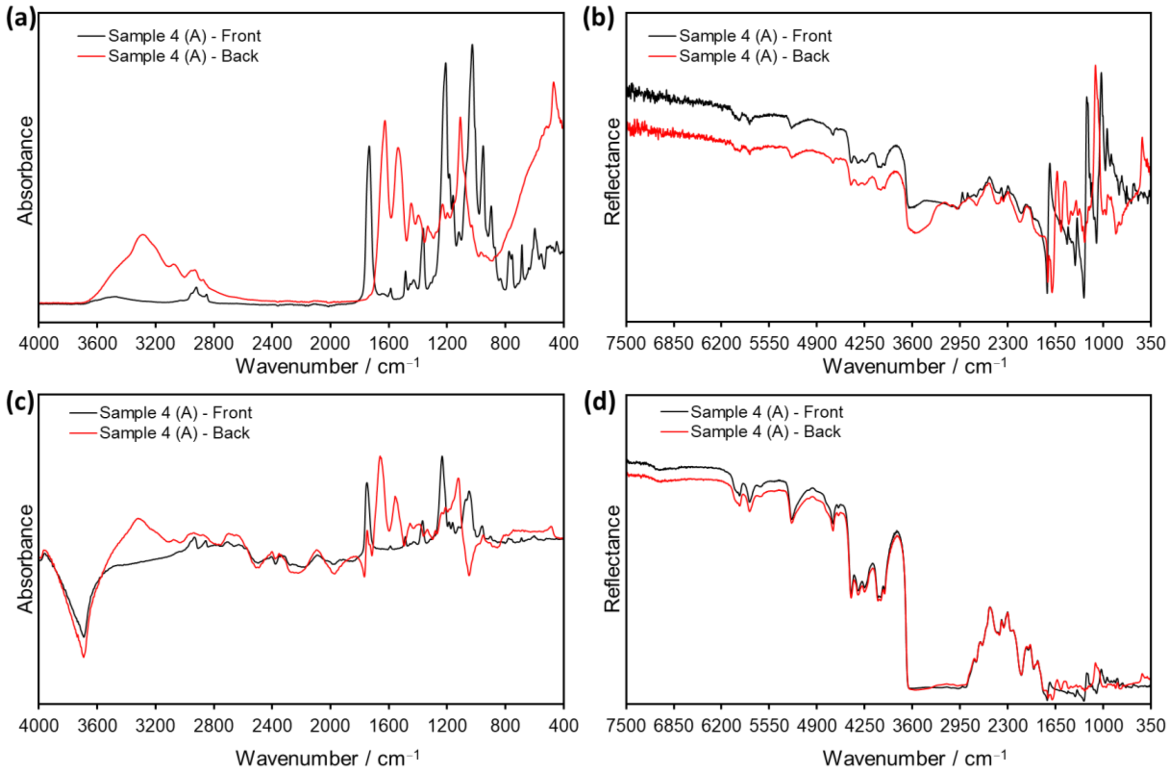

Figure S8. FTIR spectra of Sample 4 (cellulose acetate) recorded on the front side (black) and the back side (red) of the film: (**a**) ATR; (**b**) reflection; (**c**) KK transformation of reflection curves; (**d**) transflection.

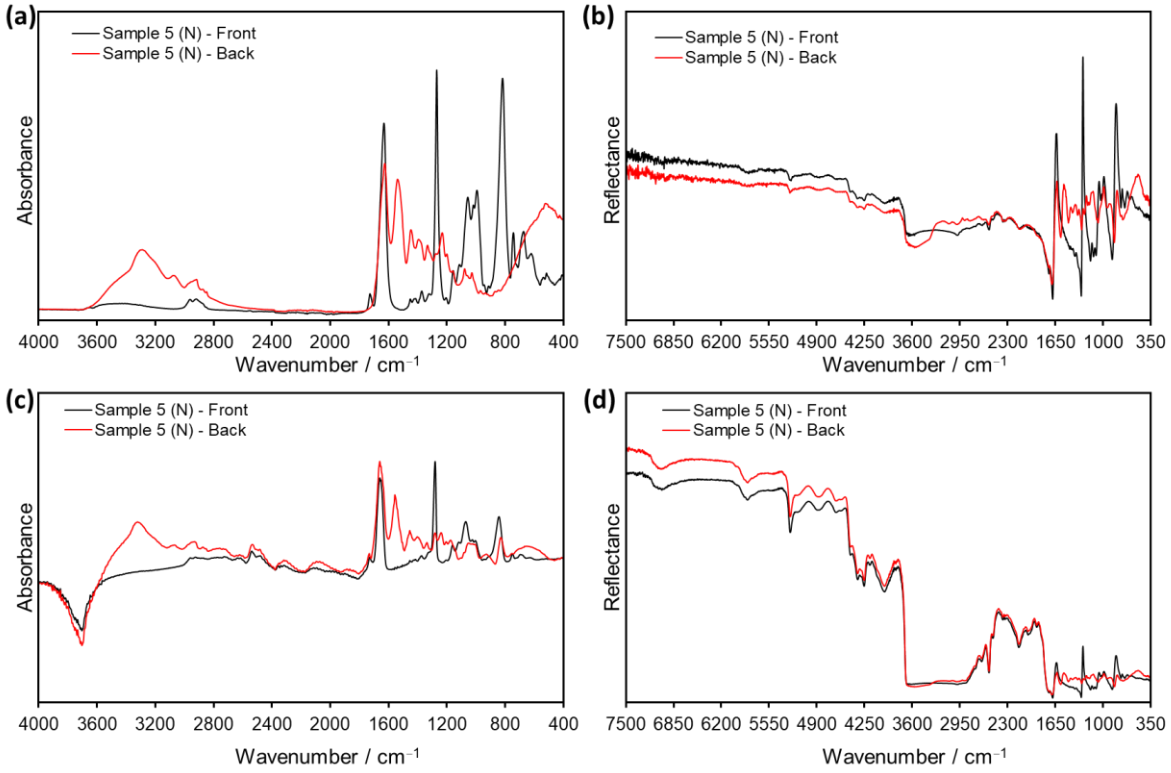

Figure S9. FTIR spectra of Sample 5 (cellulose nitrate) recorded on the front side (black) and the back side (red) of the film: (**a**) ATR; (**b**) reflection; (**c**) KK transformation of reflection curves; (**d**) transflection.

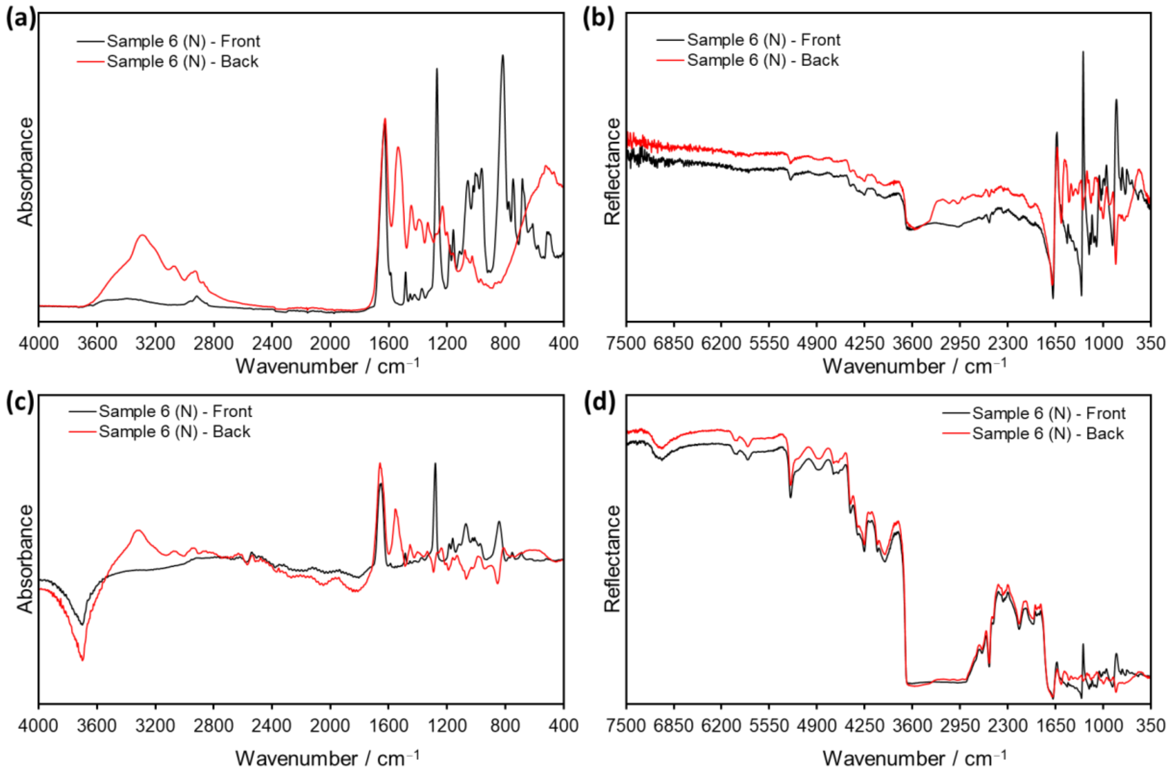

Figure S10. FTIR spectra of Sample 6 (cellulose nitrate) recorded on the front side (black) and the back side (red) of the film: (**a**) ATR; (**b**) reflection; (**c**) KK transformation of reflection curves; (**d**) transflection.

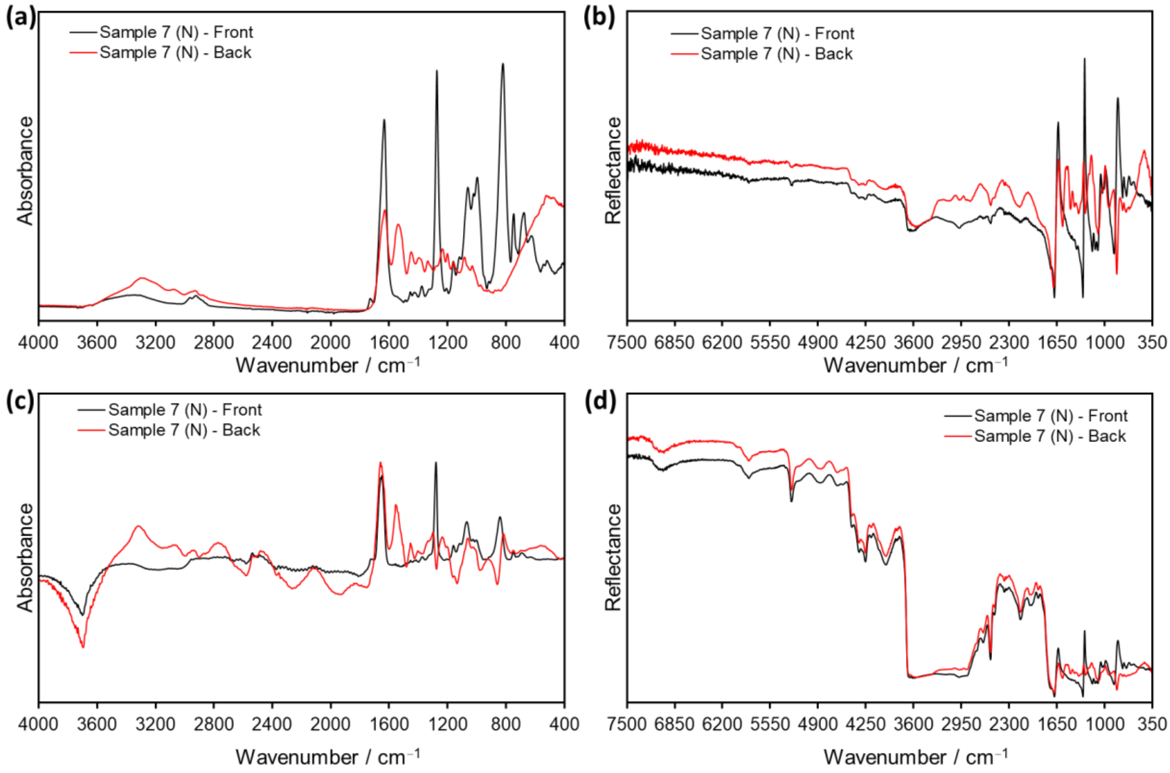

Figure S11. FTIR spectra of Sample 7 (cellulose nitrate) recorded on the front side (black) and the back side (red) of the film: (**a**) ATR; (**b**) reflection; (**c**) KK transformation of reflection curves; (**d**) transflection.

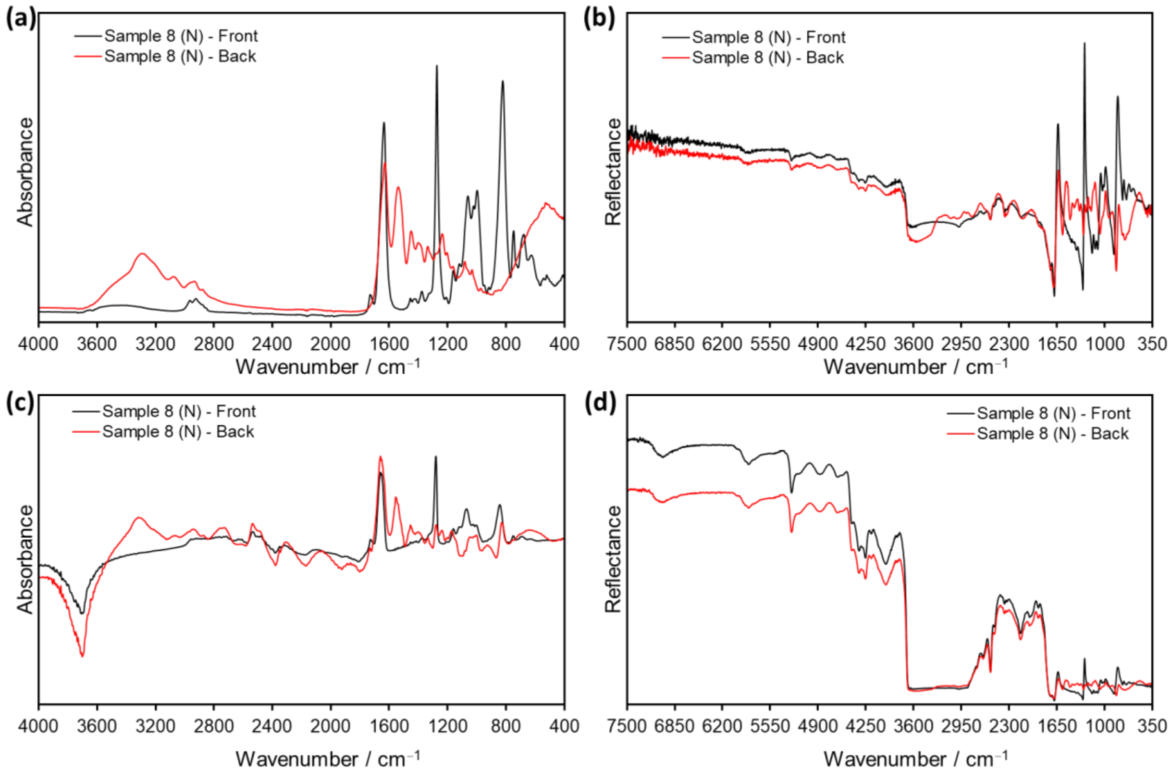

Figure S12. FTIR spectra of Sample 8 (cellulose nitrate) recorded on the front side (black) and the back side (red) of the film: (**a**) ATR; (**b**) reflection; (**c**) KK transformation of reflection curves; (**d**) transflection.

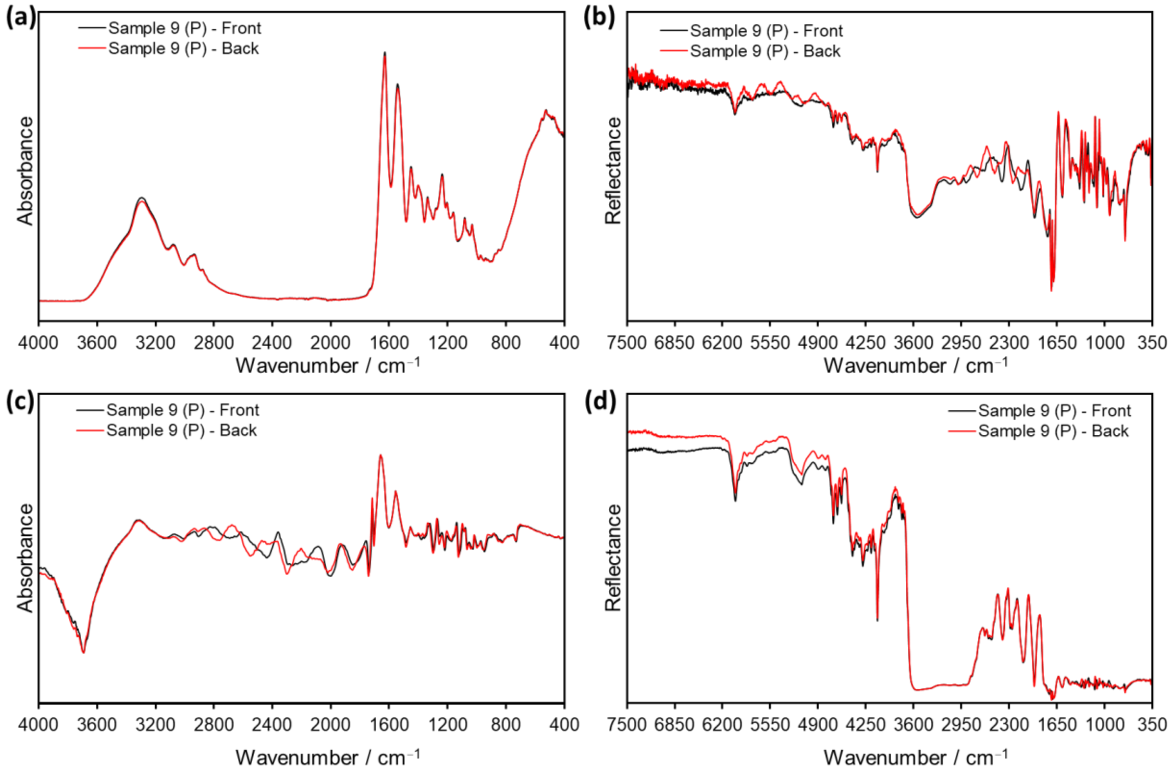

Figure S13. FTIR spectra of Sample 9 (polyester) recorded on the front side (black) and the back side (red) of the film: (**a**) ATR; (**b**) reflection; (**c**) KK transformation of reflection curves; (**d**) transflection.

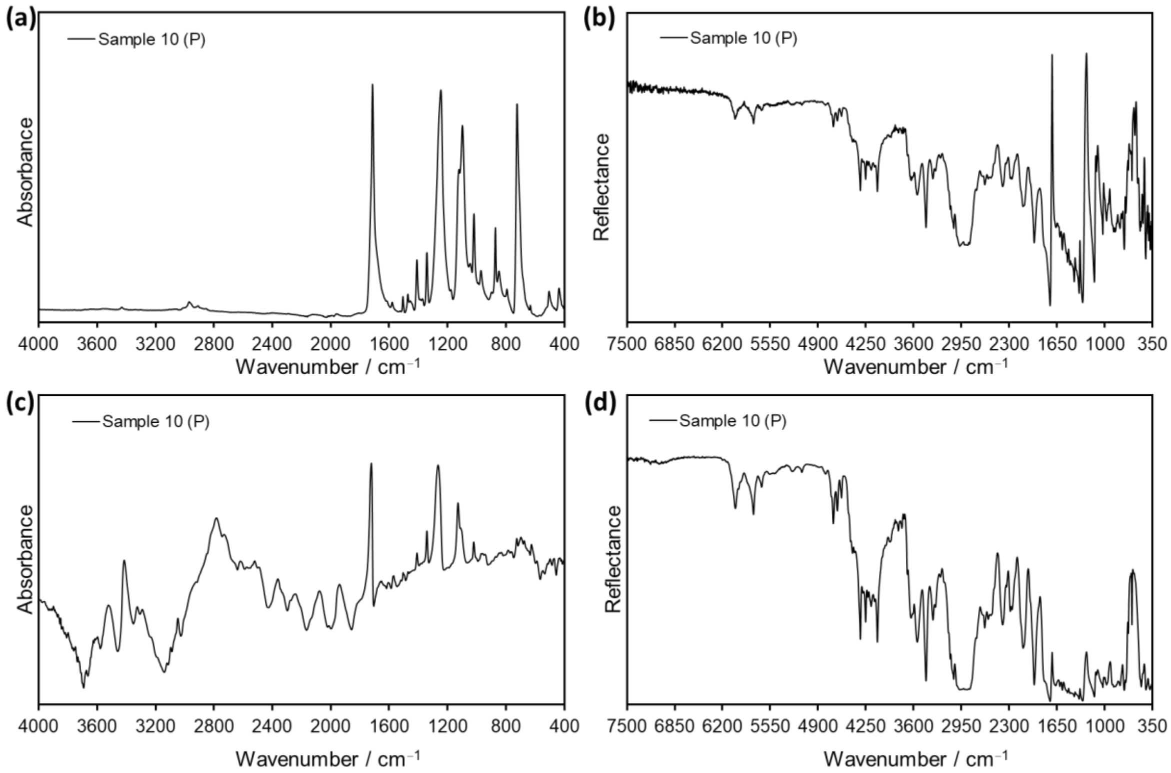

Figure S14. FTIR spectra of commercial PET sleeve (polyester), Sample 10: (**a**) ATR; (**b**) reflection; (**c**) KK transformation of reflection curve; (**d**) transflection.

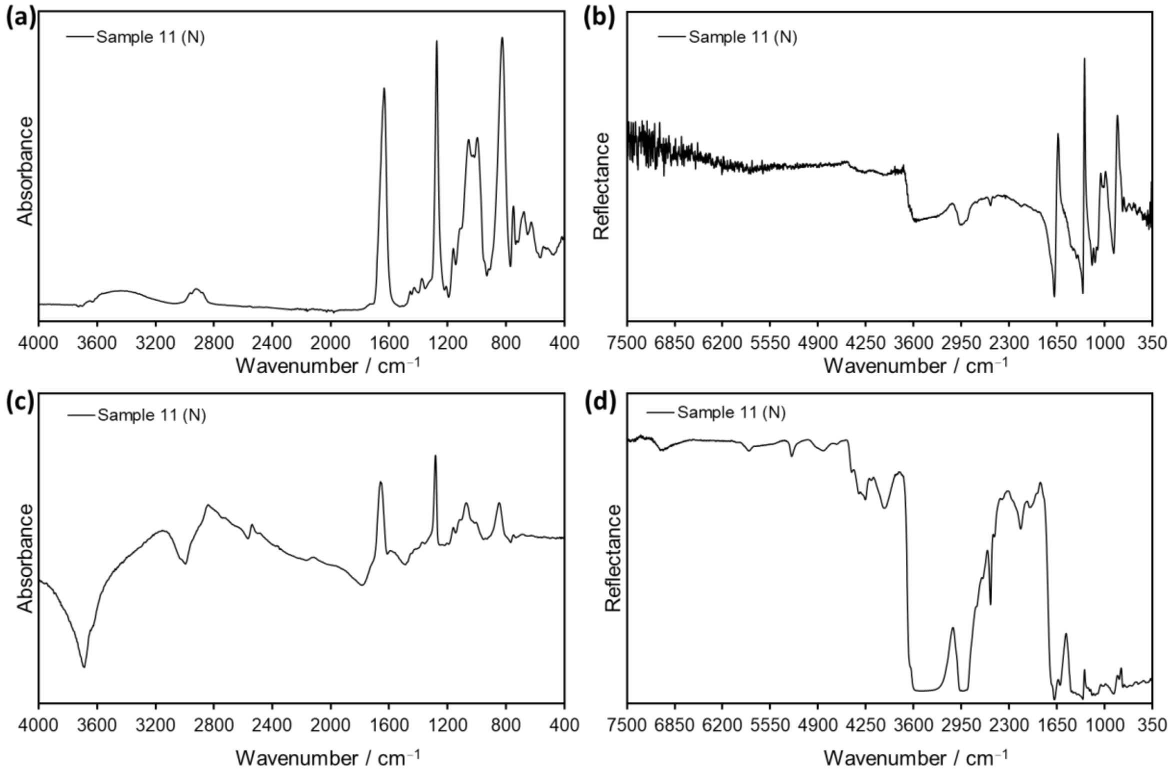

Figure S15. FTIR spectra of commercial Zapon lacquer film (cellulose nitrate), Sample 11: (**a**) ATR; (**b**) reflection; (**c**) KK transformation of reflection curve; (**d**) transflection.
